# Construction of *in vitro*–*in vivo* correlation (IVIVC) degradation evaluation model of absorbable PCL/β-TCP alveolar bone repair scaffold and exploration of a biosafety evaluation idea based on “homeostasis”

**DOI:** 10.1093/rb/rbag176

**Published:** 2026-08-14

**Authors:** Meng Zhong, Zhiqiang Zhou, Changmei Niu, Xi Yang, Yang Li, Sheng Ye, Panxianzhi Ni, Tun Yuan

**Affiliations:** National Engineering Research Center for Biomaterials, Sichuan University, Chengdu 610064, China; College of Biomedical Engineering, Sichuan University, Chengdu 610064, China; Novaprint Therapeutics Suzhou Co, Ltd., Suzhou 215000, China; Novaprint Therapeutics Suzhou Co, Ltd., Suzhou 215000, China; Novaprint Therapeutics Suzhou Co, Ltd., Suzhou 215000, China; State Key Laboratory of Oral Diseases & National Centre for Stomatology & National Clinical Research Centre for Oral Diseases & Department of Oral and Maxillofacial Surgery,West China Hospital of Stomatology, Sichuan University, Chengdu 610064, China; National Engineering Research Center for Biomaterials, Sichuan University, Chengdu 610064, China; School of Big Health & Intelligent Engineering, Chengdu Medical College, Chengdu 610064, China; National Engineering Research Center for Biomaterials, Sichuan University, Chengdu 610064, China; National Engineering Research Center for Biomaterials, Sichuan University, Chengdu 610064, China; College of Biomedical Engineering, Sichuan University, Chengdu 610064, China; Sichuan Testing Center for Biomaterials and Medical Devices Co., Ltd, Chengdu 610064, China

**Keywords:** resorbable bone repair scaffold, degradation assessment, biomaterials evaluation, tissue-response homeostasis

## Abstract

Traditional *in vivo* degradation assessments for resorbable medical devices face challenges due to extreme durations, high costs and a lack of criteria for reaching tissue-response steady states. This study investigates a 3D-printed PCL/β-TCP bone grafting scaffold by designing a model that correlates multi-stage *in vitro* accelerated degradation with *in vivo* implantation across a full 25-month timeline. This integrated approach allows for a systematic evaluation of safety and efficacy by overlapping key degradation stages. Results identified a distinct three-phase degradation pattern; the scaffold provided robust bone regeneration and maintained mechanical support for 18 months, exhibiting excellent osseointegration and biocompatibility throughout. Significantly, the correlation model identified a ‘risk peak’ regarding safety and functionality during degradation. By utilizing this concept to evaluate tissue compatibility, the study provides the experimental evidence necessary to establish ‘tissue-response homeostasis’. Ultimately, this research proposes a novel evaluation strategy rooted in homeostasis principles, significantly improving the reliability of long-term degradation predictions. It establishes an efficient performance validation pathway for clinical scenarios like alveolar bone repair and offers vital guidance for the development of regulatory systems for similar resorbable medical devices.

## Graphical abstract

**Graphical Abstract:**
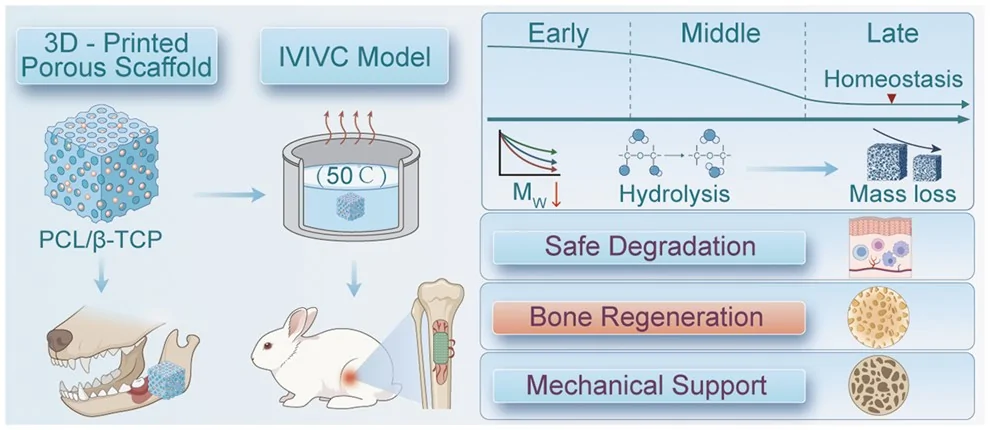

## Introduction

In recent years, with the widespread adoption and advancement of oral implant restoration techniques, functional bone regeneration—such as repair of maxillofacial bone defects and alveolar bone atrophy—has become a major clinical challenge. An ideal bone-repair material should not only possess excellent biocompatibility, but also achieve a dynamic balance among degradation characteristics, mechanical support and osteoinductive activity, thereby guiding an orderly bone-regeneration process [1–3].

Currently, bone graft materials commonly used in clinical practice—including xenografts, allografts and conventional synthetic materials—still exhibit limitations in mechanical strength, long-term structural stability and controllable degradation [4–7]. Additive manufacturing (3D printing) has enabled precise control of both the macroscopic geometry and the microscopic porous architecture of scaffolds, creating an ideal microenvironment for cell growth, nutrient transport and vascular ingrowth. These advantages allow the scaffold to better match the complex anatomical shape of defects such as the alveolar socket, showing unique clinical potential [6, 8–11].

Against this backdrop, 3D-printed tissue-engineering scaffolds composed of degradable polymers and bioactive ceramics—particularly polycaprolactone (PCL) combined with β-tricalcium phosphate (β-TCP)—have attracted significant attention due to their tunable degradation rate and osteogenic potential. These composite scaffolds not only offer improved mechanical properties but also enhance surface hydrophilicity, thereby promoting osteoblast adhesion and proliferation [12–14]. The *in vivo* degradation of PCL is a dynamic, continuous process driven by both hydrolysis and enzymatic catalysis and complete clearance relies on cell-mediated metabolic pathways. The introduction of β-TCP significantly modulates PCL’s degradation behavior by enhancing hydrophilicity and interfacial hydrolysis, effectively accelerating degradation and enabling the degradation period to better match the timeline of bone healing (Figure 1) [15–18]. Several similar devices have already been used clinically for years. For example, Woodruff *et al.* applied a 3D-printed PCL/TCP composite scaffold to repair a large cranial defect in a 9-year-old girl. Significant bone mineralization was observed at 6 months post-implantation, and by 24 months the scaffold was completely replaced by newly formed bone, with no adverse clinical events reported [19]. George *et al.* used a PCL/TCP scaffold as a structural framework for guided bone regeneration while incorporating a vascularized autologous periosteum flap, ultimately reconstructing a segmental bone defect as long as 36 cm. This technique was named Regenerative Matching Axial Vascularization (RMAV) [20].

**Figure 1:**
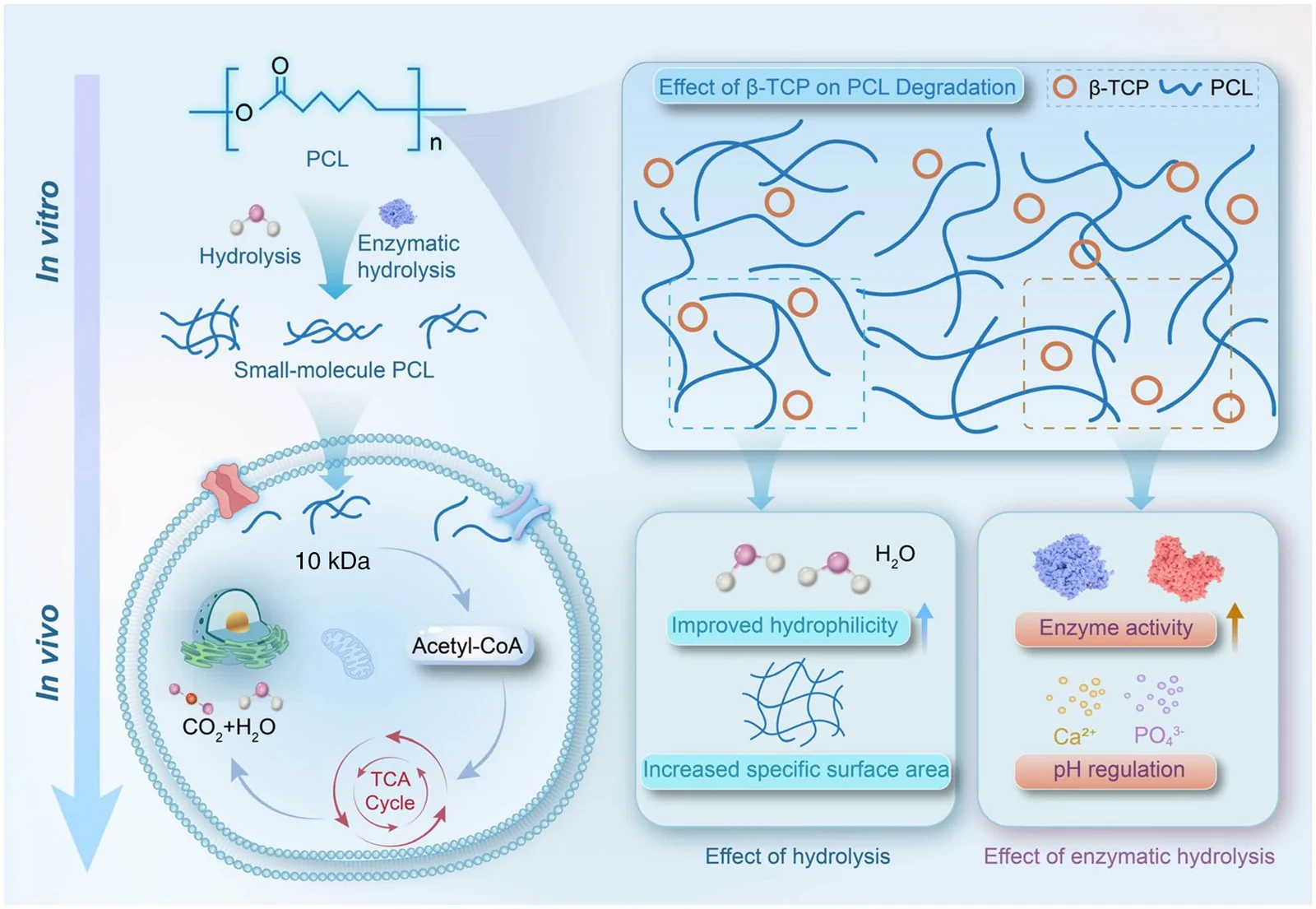
Schematic illustration of the degradation mechanism of PCL and the influence of β-TCP on PCL degradation.

**Figure 2:**
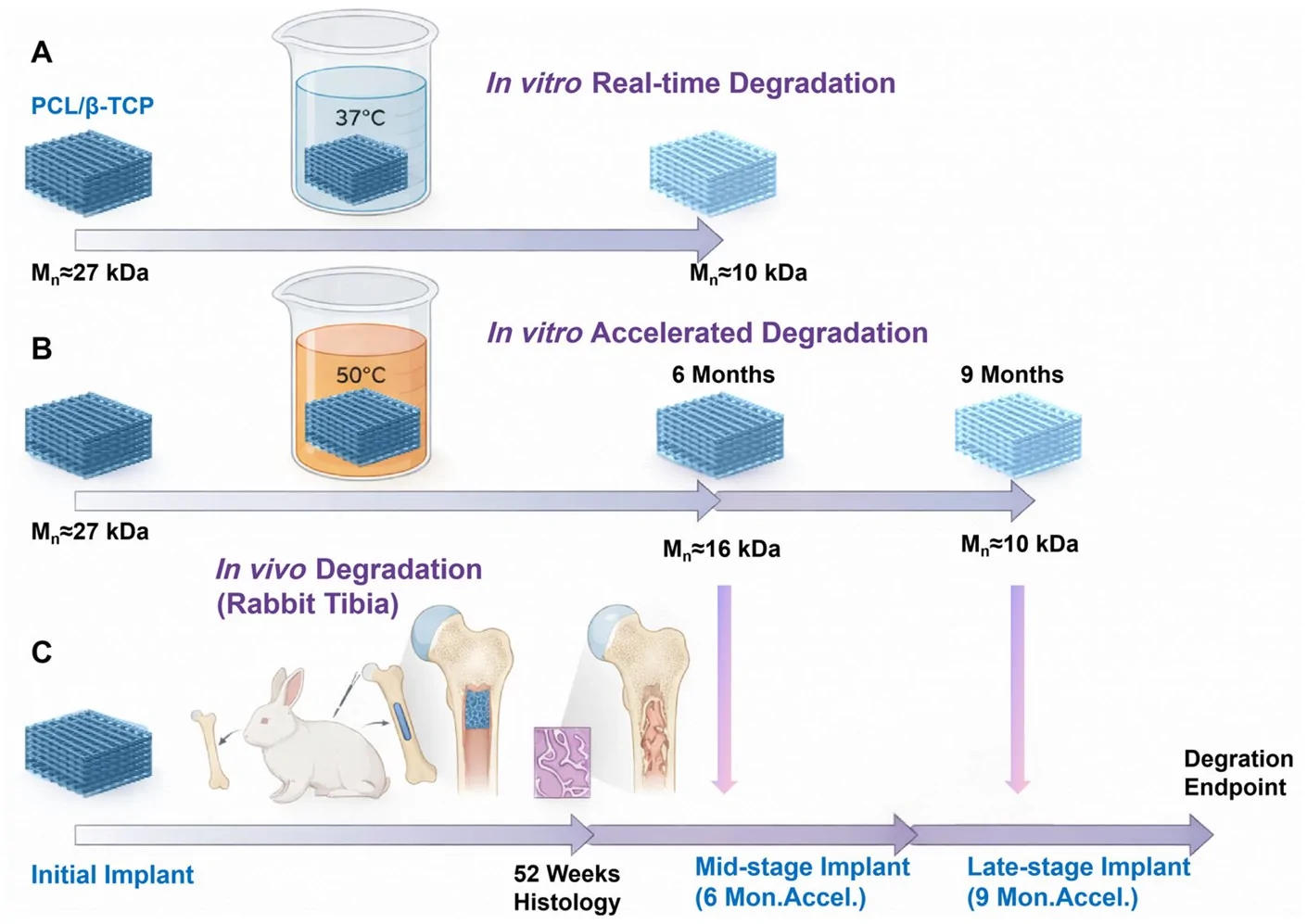
Overall design of degradation study combining *in* *vitro* and *in* *vivo* evaluation.

Despite significant progress in material design and manufacturing technologies, systematic evaluation of long-term degradation behavior and biosafety of resorbable bone-repair materials remains highly challenging. First, because the complete degradation cycle of such devices typically lasts 24–36 months, conventional *in vivo* studies require extensive time and resources, severely slowing research and regulatory review processes [21, 22]. Second, existing *in vitro* degradation models are often overly simplified and fail to accurately reproduce the complex and dynamic physiological environment *in vivo*—including cellular involvement, mechanical loading and inflammatory responses—raising concerns regarding their reliability in predicting long-term *in vivo* behavior [22]. More importantly, resorbable composite biomaterials undergo continuous interactions with the host, and without a systematic framework that captures the dynamic evolution at the material–tissue interface throughout the full degradation process, regulatory evaluation lacks scientific criteria for identifying safety- and efficacy-related ‘risk peaks’ or for determining when tissue response reaches homeostasis [23].

These limitations greatly hinder the clinical translation of innovative products. Taking alveolar socket preservation as an example, clinicians urgently need reliable methods to predict degradation behavior and bone-regeneration outcomes within 1–3 years after implantation; however, the current evaluation systems fall short of this requirement. Additionally, for bioactive composites containing inorganic phases such as calcium phosphate ceramics, their altered physicochemical properties further change the mechanisms of interaction with biological tissue. Regulatory agencies currently lack appropriate tools and scientific standards for evaluating such novel materials, leading to extended review timelines that delay the adoption of promising innovations [24].

To systematically address these issues, this study aims to establish an innovative evaluation framework focused on resolving three core challenges: first, to investigate the degradation mechanisms and long-term biosafety of PCL/β-TCP composite scaffolds, elucidating their degradation behavior under complex physiological conditions; second, to establish a quantitative correlation between real-time *in vitro* degradation and accelerated degradation models, thereby validating the predictive efficacy of *in vivo* experiments based on accelerated-aging samples; and finally, to explore a definition of ‘homeostatic’ tissue responses and a corresponding risk assessment method suitable for resorbable medical devices, providing scientific evidence to support regulatory evaluation.

In this study, we fabricated PCL/β-TCP composite scaffolds with precisely controlled three-dimensional porous structures using additive manufacturing to ensure sample consistency and comparability. Through accelerated hydrolysis experiment *in vitro*, the effect of β-TCP on the degradation of PCL was systematically verified. It was proved that β-TCP improved the overall degradation efficiency without changing the chemical degradation mechanism of PCL. We then developed an innovative evaluation model integrating multi-stage *in vitro* accelerated degradation with *in vivo* validation. By implanting samples pre-aged for different durations under accelerated *in vitro* conditions, this model achieved full coverage of the entire degradation lifecycle and allowed cross-validation of key stages. Beyond significantly shortening the evaluation timeline, this approach establishes a direct correspondence between *in vitro* and *in vivo* degradation behaviors, providing a robust scientific foundation for predictive modeling. The results demonstrate that the composite scaffold exhibits a clear three-phase degradation pattern, highly predictable long-term behavior, excellent biosafety and reliable osteogenic efficacy, with its total degradation cycle well-matched to the timeline of bone regeneration. Notably, accelerated-aged samples yielded *in vivo* results highly consistent with long-term implantation data, confirming the reliability and predictive value of the evaluation strategy.

For bone implants or oral products made of absorbable synthetic polymer materials with a long degradation cycle, how to set the degradation observation end point has always been the key to the formulation of relevant industry standards [25, 26]. The *in vivo* degradation part should focus on the study of safety and effectiveness. Taking the alveolar bone repair stent as an example, we believe that its safety qualification index means that the tissue reaction does not reach the stimulation threshold during the whole implantation cycle, and there is no unacceptable toxic reaction or foreign body rejection, while the effectiveness qualification index means that the material can provide good shape and mechanical support within the expected design period, and promote the formation of blood vessels and new bones [27]. Existing standards require that *in vivo* degradation studies continue until the implant is nearly resorbed or until tissue response stabilizes—i.e. until ‘homeostasis’ is reached. The definition of homeostasis needs to take the safety and effectiveness indicators as an important reference.

This study highlights the concept of the ‘risk peak’ during the degradation process of resorbable devices—i.e. the period when safety and efficacy risks are highest. If the device has already fulfilled functional requirements and tissue reaction remains within acceptable limits before the risk peak, and if both parameters are expected not to worsen thereafter, the material–tissue system may be considered to have reached homeostasis even before complete degradation. This insight may help shorten regulatory evaluation timelines [21, 28]. The findings presented here not only guide product development but also provide a methodological foundation for establishing regulatory science frameworks for evaluating resorbable medical devices.

## Materials and methods

### Materials

The PCL/β-TCP composite bone grafting material used in this study was provided by Novaprint Therapeutics Suzhou Co., Ltd. (Suzhou, China). The scaffolds were fabricated from a biphasic composite consisting of 80% PCL and 20% β-TCP using a melt-microextrusion additive manufacturing process, yielding a structure biomimetic to natural bone. Three sample types were prepared: compression specimens, mesh-type specimens and tibial implantation samples. The compression specimens measured 5.4 × 10 × 10 mm, meeting the dimensional requirements for standard compression testing. The mesh-type specimens measured 50 × 25 × 1.3 mm, representing a typical clinical configuration and fulfilling bending-test requirements. The tibial implantation samples measured φ2 × 6 mm, satisfying the dimensional specifications for bone implantation biocompatibility studies. For each time point, five standard compression specimens and five mesh specimens were tested, with each sample placed in an individual container. One blank control sample was included for each time point.

### Degradation study design

This study followed the ISO 10993-13:2010 standard to simulate hydrolytic degradation through real-time and accelerated *in vitro* tests, with qualitative and quantitative analysis of degradation products. A rabbit tibial implantation model was also employed to represent three distinct *in vivo* degradation phases. Histological and quantitative analyses were used to characterize tissue responses and material changes throughout the entire degradation process, allowing comprehensive evaluation of biodegradability and biosafety.

The overall design logic was as follows: *in vivo* studies were used to determine safety endpoints (tissue response) and confirm degradation trends in a physiological environment, while *in vitro* studies elucidated the materials science mechanisms underlying these biological changes, including morphological evolution, chemical composition, molecular weight loss, degradation product release, mechanical properties and crystallinity. Together, the *in vitro* and *in vivo* studies provided a complete understanding of degradation behavior and biocompatibility, supporting long-term clinical safety assessment.

### Effect of β-TCP on PCL degradation

PCL primarily degrades via hydrolysis of ester bonds and exhibits a relatively slow degradation rate under physiological conditions. Strong alkaline hydrolysis using high-concentration NaOH can significantly accelerate ester bond cleavage, enabling rapid assessment of degradation behavior. Standard samples were fabricated using PCL and PCL/β-TCP composite materials, and accelerated degradation experiments were conducted *in vitro* to evaluate the effects of β-TCP addition on material structure changes, mass loss, mechanical properties and organic component content variations. The degradation solution used was a 5 mol/L NaOH solution with a liquid volume (mass-to-volume ratio) of 1:100 g/mL. The degradation process was conducted at a temperature of 37°C. The observation periods for the PCL group were 0, 2, 7, 12, 15, 18 and 21 days, while for the PCL/β-TCP group, the observation periods were 0, 12, 24, 36, 48, 72 and 96 hours.

### *In vitro* degradation of the composite materials

*In vitro* studies encompass real-time degradation research: samples are immersed in PBS buffer (pH 7.4) at 37°C for a period of 42 months, simulating the hydrolysis process *in vivo*. Accelerated degradation research is conducted at 50°C for 18 months to swiftly ascertain degradation trends. The selection of 50°C was strictly guided by the physicochemical properties of PCL and established ISO standards (ISO 10993-13). Samples soaked for varying durations undergo comprehensive analysis, including mass loss, microscopic morphology (SEM), chemical composition (FTIR), molecular weight changes (GPC), quantitative analysis of degradation products (HPLC), mechanical properties and crystallinity (DSC).

### Experimental study on degradation of rabbit tibia implanted in bone

In this study, a rabbit tibial bone implant model was used. The standard ISO 10993-6:2016 and ISO 10993-17:2023 was strictly followed, aiming to comprehensively evaluate the biodegradation behavior and host response of the product in the *in vivo* environment. The setting of sampling time point was based on the molecular weight change rule of the previous *in vitro* degradation study to completely cover the three typical stages of the degradation process (Table 1):

**Table 1:** Degradation cycle of tibia implantation experiment in rabbits.

	Part 1 Implantation *in vivo*					Part 2 Implanted after 6 months of *in vitro* acceleration			Part 3 Implanted after 9 months of *in vitro* acceleration				
Implantation time point (weeks)	0	4	12	26	52	0	12	26	0	1	4	12	31
Corresponding *in vivo* real-time implantation cycle (weeks)	0	4	12	26	52	52	64	78	78	79	82	90	109

The first stage: this stage covered the early process from the beginning of implantation to the significant decline of molecular weight. The endpoint was set according to the results of the *in vitro* studies.

The second stage: the starting point and end point of this stage were set according to the degradation characteristics obtained by accelerated degradation *in vitro*. The starting point corresponded to the middle of the rapid molecular weight decline period in the *in vitro* study, and the end point was close to the time point at which the molecular weight threshold (below 10 kDa) at which cell-mediated clearance began to be active *in vivo*. This stage is a period when the mechanical properties of products had changed significantly and mass loss had begun to occur.

The third stage: the material taken at this stage corresponded to the slowing down of the molecular weight decline rate in the *in vitro* study, and the molecular weight had decreased to the beginning of cell clearance (i.e. below the 10 kDa threshold), and continued until the implant is completely fragmented and finally cleared by the body or close to the final clearance. The setting of this phase was directly based on the molecular weight threshold of the clear *in vitro* study and the initiation of cell clearance mechanism, which was used to focus on the evaluation of the final biological clearance process and tissue reaction of the material at low molecular weight.

The core evaluation indicators of *in vivo* research include: analyzing the degradation rate through volume change, and evaluating the local tissue response according to the compliant histopathological scoring system. In order to ensure the accuracy of histological evaluation, the micro morphology of the removed implants was not analyzed in the *in vivo* experiment to avoid damage to the tissue structure caused by the sample processing process.

The experimental steps were as follows: a longitudinal incision (∼ 3–5 cm) was achieved in the area of the medial tibial shaft (or other appropriate sites) followed by a step-by-step separation to expose the cortex of each tibia, to separate the periosteum and to try to protect the periosteum. On the tibia, preferably the medial side, drill three holes (proximal, middle and distal) ∼ 0.5–1.0 cm apart for receiving the implants. Ream to final diameter before implantation and wash the wound with physiological saline. On one side, insert the test articles by finger or tool pressure to allow press-fit, with the implanted samples levelled. Animals of group 2 were implanted in the same location and of groups 1 and 3 were implanted with a control article in the same location (∼0.06 g per implant site). The incisions were closed with sutures and/or skin staples as needed, taking precautions to maintain aseptic conditions. Aluminum wound spray was applied onto the closed skin incisions to protect the wound.

Under different implantation cycles, the diameter of the remaining implants was measured under the optical microscope to analyze the residual amount of implants. Only the similar circular section was taken as the statistical plane for measurement. Taking the diameter of the different starting points of the implants as the initial amount, the degradation rate of implants was calculated according to formula (1):


(1) $$D=(1-\frac{r^{2}}{R^{2}})\times 100\%$$

(D: Implant degradation rate; r: Residual diameter of implants; R: Initial implant diameter).

Histopathological evaluation was performed by pathologists in accordance with standard operating procedures (SOPs), ISO 10993-6:2016 and ISO 10993-17:2023. The local cellular and tissue responses at the implant/tissue interface were evaluated and recorded using a semi quantitative evaluation system.

The bone push-out test was used to detect the adhesion between the sample and bone after different implantation cycles. The sample size was φ 2 **×** 6 mm. The PCL/β-TCP sample was implanted into the experimental group, and the normal bone tissue of rabbit tibia was implanted in the control group. The implantation cycles were 11, 24, 36 and 48 weeks, respectively. After the tissue block implanted in the tibia was taken out, ensured that the test surface of the sample was flat and the plane of the implant was completely perpendicular to the test thimble, and drew a circle with a diameter of about 2 mm on the bone surface with a marker pen through the strong light. The normal bone tissue-like blocks around the implants were tested synchronously on the same tibia. The 200 N pressure sensor was used to make the implant plane completely perpendicular to the thimble, and the porous surface of the sample was compressed. The compression speed was set to 5 mm/min until the sample was pushed out. The relative bone implant bonding strength (expressed as a percentage of normal rabbit tibia tissue) was calculated at different cycles. Bone implant relative bonding strength = (maximum push out force of experimental group/maximum push out force of normal rabbit tibia tissue in control group) **×**100%.

### Experiment of canine oral cavity restoration

The size of PCL/β-TCP sample in the dog oral defect repair experiment was φ 14 **×** 2 mm, and the control group was treated with Bio-Oss powder (Geistlich Pharma AG). Twelve male beagles aged about 2 years and weighing 12–17 kg were randomly divided into the experimental group and the control group. Samples were taken at 12 weeks and 26 weeks, with 3 dogs in each group. The model was made in the mandibular third premolar. The operation steps were as follows: Sumianxin + pentobarbital sodium combined anesthesia, venous indwelling needle to establish venous access. Lidocaine hydrochloride was injected into the gingiva after tooth extraction for local anesthesia. The third molar was extracted after the canine tooth was extracted from the mandible. The left and right sides were treated the same. The gingiva was sutured with absorbable suture. Analgesia and anti-infection were performed every day for 3 days after operation. Subcutaneous injection of meloxicam at 0.2 mg/kg was used for analgesia, and intramuscular injection of ceftiofur sodium (20 mg/mL) 0.1 mL/kg was used for anti-infection. The wound healing was checked every day. Two weeks later, the second operation was performed. After anesthesia, a 1.5-cm incision was made to the top of the alveolar ridge in the middle of the alveolar ridge, and the full-thickness mucoperiosteal flap was turned up to expose the alveolar bone. The defect was prepared with a drill bit, and the defect volume was about 10 × 4 × 4 mm. The sample package was opened in a sterile environment, the sample was filled into the left defect of the dog, and the skin incision was sutured with absorbable suture. The control substance was filled into the right defect of the dog, and the skin incision was sutured with absorbable suture. Analgesia and anti-infection were performed every day for 3 days after operation.

Micro CT was used to analyze the new bone formation. The scanning parameters were set to 11.4 μm pixels, 70 kV voltage, 200 μA current and 0.12° rotation step. After scanning, Mimics software was used to reconstruct the image. For the oral implant area, a cube area with a length of 10 mm, a width of 3.5 mm and a height of 3.5 mm was selected around the defect area as the volume of interest, and then bone mineral density (BMD), bone volume fraction (BV/TV), number of trabeculae (Tb.N) and thickness (Tb.Th) were further analyzed. After the materials were taken from the modeling position, they were fixed in 10% buffered formalin solution for 72 hours, and then dehydrated with gradient ethanol (treated with 70% ethanol, 95% ethanol, 100% ethanol, 1:1 ethanol acetone mixture and 100% acetone in turn). After dehydration, they were embedded with Spurr resin. A low-speed diamond saw (rotating speed 300 rpm) was used to cut the embedded sample along the parallel direction of the annular section of the implant, and the slices were bonded with glass slides and then ground to prepare thin slices. Finally, the biological safety and osteogenic effect after implantation were observed and analyzed by H&E staining and Goldner trichrome staining under the light microscope.

### Statistical analysis

All data were expressed as means ± standard deviation of three representative experiments. The normal distribution analysis was checked by the Shapiro-Wilk test and quantile-quantile. The homogeneity of variances was tested by the Brown-Forsythe test. Parametric statistics were conducted using GraphPad Prism software (GraphPad Software Inc.) by Student’s *t*-test (unpaired and two-tailed) and one-way ANOVA, followed by Tukey *post hoc* test. The values were considered significantly different at *P *< 0.05.

## Results

### Effect of β-TCP on PCL degradation

A multidimensional characterization of the degradation behavior of both PCL and PCL/β-TCP groups was performed, including SEM morphological observation, mass-loss measurement, mechanical-property evaluation and FTIR spectroscopy. SEM imaging revealed that β-TCP particles gradually detached during degradation, forming microporous structures and increasing the specific surface area of the PCL matrix, thereby accelerating hydrolysis and enhancing the overall degradation efficiency (Figure 3A).

Comparison of mass-loss curves and mechanical-property changes under identical degradation conditions showed that both groups exhibited gradual reductions in mass and mechanical strength with increasing degradation time (Figure 3B–D). The PCL/β-TCP group reached 95.59% mass loss within 96 hours, whereas the PCL group required 21 days to achieve 96.43% mass loss, confirming that β-TCP significantly accelerates the degradation of PCL.

FTIR analysis demonstrated that characteristic absorption peaks at 1168 cm⁻¹, 1721 cm⁻¹, 2858 cm⁻¹ and 2945 cm⁻¹ remained unchanged in both the PCL/β-TCP group after 72 hours of degradation in 5 mol/L NaOH and the PCL group after 18 days of degradation, indicating that no chemical structure alteration occurred during the degradation process. The ester-bond hydrolysis mechanism of the PCL matrix remained intact (Figure 3E).

### Validation of the relationship between *in vitro* real-time and accelerated degradation

The physical and chemical characterization results of the sample after real-time degradation and accelerated degradation *in vitro* are shown in Figure 4. The composite scaffold product follows the degradation law of PCL products, which is a hydrolysis process from outside to inside, and its surface also shows the phenomenon of gradual roughness. However, due to the absence of enzymatic hydrolysis and metabolic discharge processes *in vitro*, the overall morphology changes slowly (Figure 4A). From the infrared absorption spectra of accelerated and real-time degradation *in vitro*, there was no significant change in the chemical functional groups of the whole process (Figure 4B). The degradation of the samples followed the classical three-stage degradation kinetics of PCL, that is, a rapid decline period (corresponding to 0–3 months of acceleration *in vitro*): the number average molecular weight (M_n_) decreased rapidly from 26 638 Da to 17 987 Da, which was dominated by the rapid hydrolysis of ester bonds in the amorphous region; Second stage linear deceleration period (corresponding to 3–9 months of *in vitro* acceleration): M_n_ decreased from 17 987 Da to 9251 Da, and the rate was relatively slow, indicating that the hydrolysis reaction entered the crystallization zone and was controlled by diffusion; Third stage platform transition period (corresponding to 9–18 months of *in vitro* acceleration): M_n_ decreased from 9251 Da to 4927 Da, and the change tended to be gentle at this low molecular weight level. Especially, at the ninth month, M_n_ had dropped to 9251 Da, breaking the critical threshold of 10 kDa phagocytic clearance, indicating that the material was about to enter the final stage dominated by body erosion and mass loss. In addition, the consistency of the trends of real-time degradation and accelerated degradation (such as the state of accelerated 6 months M_n_=16 551 Da ≈ real-time 18 months M_n_=17 932 Da) strongly verified the effectiveness of the accelerated model and provided a core scientific basis for the strategy of using accelerated aging samples for *in vivo* experiments to predict long-term behavior (Figure 4C).

Under accelerated conditions, compressive strength remained stable in the first 6 months (–1.32% change) and retained 74.68% of its initial value at 9 months. Real-time degradation showed even greater stability (Figure 4D). These results demonstrate that the scaffold maintains ≥70% compressive strength well beyond 6 months (up to 9–18 months), meeting the design requirement of ≤30% reduction during the functional period. Flexural properties were more sensitive to molecular-weight loss. Flexural strength decreased by 33.63% at 3 months under accelerated aging and by 28.32% at 6 months under real-time conditions, followed by a slower decline. The 6-month time point corresponds to the clinically critical window during which the scaffold successfully provides adequate flexural support (Figure 4E).

Crystallinity increased gradually during both real-time and accelerated degradation due to preferential degradation of amorphous regions (Figure 4F). Overall mass loss remained low; even after 18 months of accelerated degradation, cumulative mass loss remained below 10% (Figure 4G). Finally, in the process of accelerated and real-time degradation of composite scaffold products *in vitro*, the release of caprolactone monomer, the degradation product, remained at a very low level (<10 ppm) and did not show a continuous accumulation trend, indicating that the degradation process was stable and controllable, with good biological safety and met the relevant requirements for the safety of absorbable degradation products in ISO 10993-17:2023 (Figure 4H). During the degradation process, there was no significant change or difference in pH value, indicating that accelerated degradation did not affect the erosion mechanism of PCL and the release of β-TCP (Figure 4I).

### Degradation results of three stages of rabbit tibial bone implantation experiment

The stage of *in vivo* degradation research is divided into three stages, which are directly derived from the key nodes of the above *in vitro* data: the first stage: corresponding to the rapid decline stage *in vitro*. The starting point of the second stage (≈16 kDa, accelerated degradation *in vitro* for 6 months) accurately corresponded to the deceleration period *in vitro*, and through the observation time of 26 weeks, it crossed into the cell clearance stage. The starting point of the third stage was directly set as M_n_<10 kDa (≈9000 Da), corresponding to accelerated degradation *in vitro* for 9 months. The accelerated experiment did not change the chemical mechanism of degradation, but only accelerated the reaction rate, so the prediction result is highly reliable, and it is scientific to use accelerated samples for subsequent *in vivo* research.

#### Phase I *in vivo* degradation (0–52 weeks)

This stage aimed to evaluate the biocompatibility and degradation behavior of PCL/β-TCP composite scaffold products *in vivo* in the early to middle stage of implantation. The number average molecular weight of the implants decreased from the initial value to about 15 kDa, marking the end of the initial stage of degradation.

First, the initial wire diameter of the sample was measured as a reference. Quantitative analysis showed that the average wire diameter was about 219.3 μm (Figure 5A1). Histology revealed engulfed material fragments within multinucleated giant cells at all time points (4–52 weeks), confirming ongoing *in vivo* degradation. Tissue-response characteristics (fibrosis, macrophage infiltration) were similar to controls, indicating good biocompatibility. The number of giant cells was higher around the scaffold, particularly at early time points (4 and 12 weeks), consistent with physiological clearance of resorbable materials. By 52 weeks, implant edges became irregular and internal cracks appeared, demonstrating continuous degradation (Figure 5B1). Histopathological scores remained low: 2.0 at 4 weeks, 1.6 at 12 weeks, 1.5 at 26 weeks and 2.7 at 52 weeks—classified as ‘minimal or no reaction’ (Figure 5C1).

At 52 weeks, M_n_ was 15 266 Da, slightly lower than accelerated 6-month M_n_ (16 551 Da). Thus, implanting accelerated 6-month samples allowed the Phase II investigation to seamlessly overlap with Phase I molecular-weight trends.

#### Phase II *in vivo* degradation (accelerated 6-month samples implanted for 12–26 weeks)

The initial wire diameter of the sample was measured as a reference. Quantitative analysis showed that the average wire diameter of the sample after 6 months of accelerated degradation *in vitro* was about 214.0 μm (Figure 5A2). At 12 weeks after accelerated implantation *in vitro*, tissue sections showed that the implanted repair materials were visible in the bone defect area of PCL/β-TCP group, and the implanted materials were significantly degraded. Proliferative fibrous tissue with a small number of lymphocytes can be seen around the implant. A small amount of new bone trabeculae formed at the implant boundary (Figure 5B2). The pathological analysis results after 12 weeks of implantation were ‘minimal or no reaction’ (Figure 5C2).

Twenty-six weeks after accelerated implantation *in vitro*, the results of tissue sections showed that  the implanted materials were visible in the bone defect area, and the materials continued to degrade without causing adverse reactions. Trabeculae are distributed along the implant space. Active osteoblasts can be seen at the edge of the new bone around the implanted material. During the remodeling of the new bone, the new bone tissue has grown into the material and began to occupy the space vacated by the degradation of the material (Figure 5B2). The pathological analysis results after 26 weeks of implantation were no stimulation or very slight stimulation (Figure 5C2).

The number average molecular weight of the implants was 8664 after 6 months of *in vitro* acceleration and 26 weeks of *in vivo* implantation, which was highly close to the molecular weight (M_n _= 9251) after 9 months of *in vitro* acceleration, and the *in vitro* acceleration model was validated again *in vivo* to effectively simulate the degradation process *in vivo*.

#### Phase III *in vivo* degradation (accelerated 9-month samples implanted for 1–31 weeks)

The initial wire diameter of the sample was measured as a reference. Quantitative analysis showed that the average wire diameter of the sample after 9 months of *in vitro* acceleration was about 213.6 μm (Figure 5A3). Histological results showed that the PCL/β-TCP group was well surrounded by fibrous connective tissue at the initial stage of implantation (1 week), accompanied by angiogenesis and new bone trabeculae, only a few inflammatory cells were seen and no macrophages or giant cells were found, indicating that the material had initial biocompatibility. At 4 weeks, the silk-like tissue grew into the material, and the average wire diameter decreased to 185.53 ± 8.16 μm, indicating that the degradation continued. At 12 weeks after implantation, the tissue reaction showed periodic changes: macrophages and multinucleated giant cells were occasionally seen at the beginning, the inflammatory cells at the edge of the material increased slightly, and the average filament diameter was further reduced to 164.93 ± 6.19 μm, indicating that the body had initiated the active phagocytosis and clearance process of degradation products. By the end of the implantation period (31 weeks), the material had widely grown into silk reticular fibrous tissue and was accompanied by neovascularization. The average wire diameter decreased sharply to 38.41 ± 17.25 μm, and the standard deviation increased, indicating that the material had been fragmented into fragments of different sizes, and only a few inflammatory cells were seen, suggesting that the clearance reaction was nearly completed (Figure 5B3).

**Figure 3:**
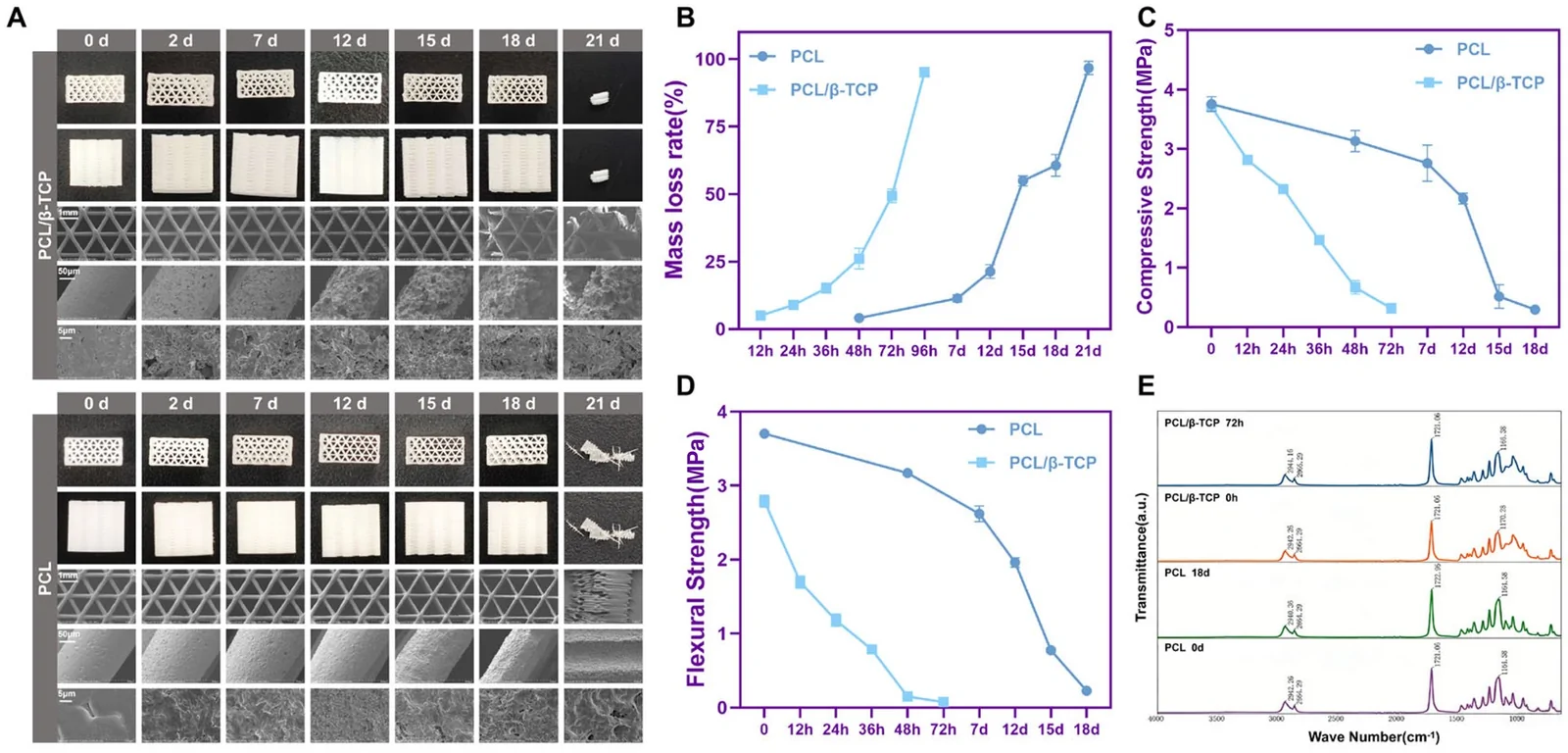
Degradation performance study results. (**A**) Macroscopic and microscopic morphology at various degradation time points. (**B**) Mass loss rate. (**C**) Flexural strength. (**D**) Compressive strength. (**E**) FTIR spectra before and after degradation.

**Figure 4:**
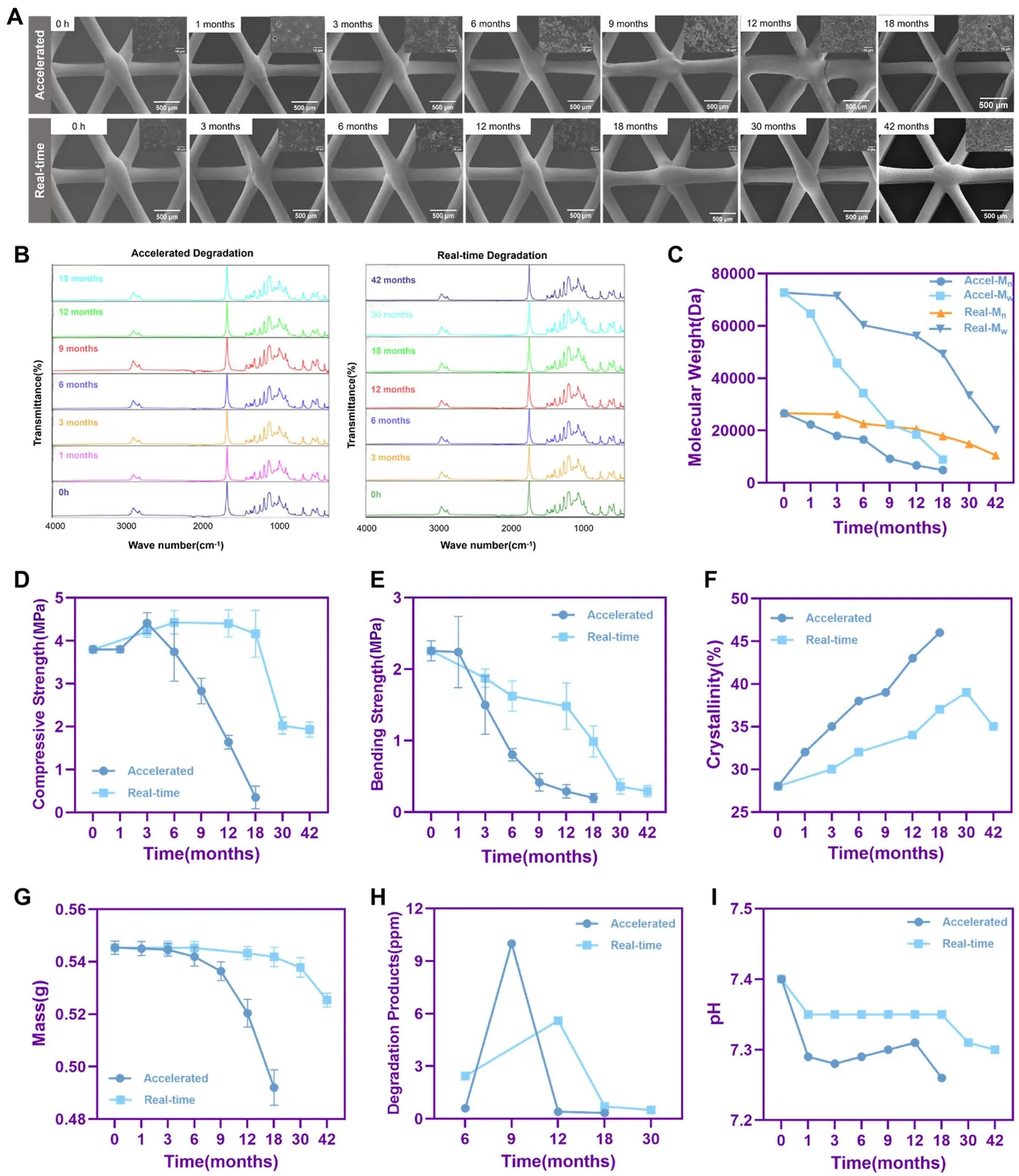
Analysis of *in vitro* degradation characteristics under different degradation conditions. (**A**) SEM morphology. (**B**) FTIR spectra. (**C**) Molecular-weight evolution. (**D**) Compressive strength. (**E**) Flexural strength. (**F**) Crystallinity. (**G**) Mass loss. (**H**) Quantification of degradation products. (**I**) pH value.

**Figure 5:**
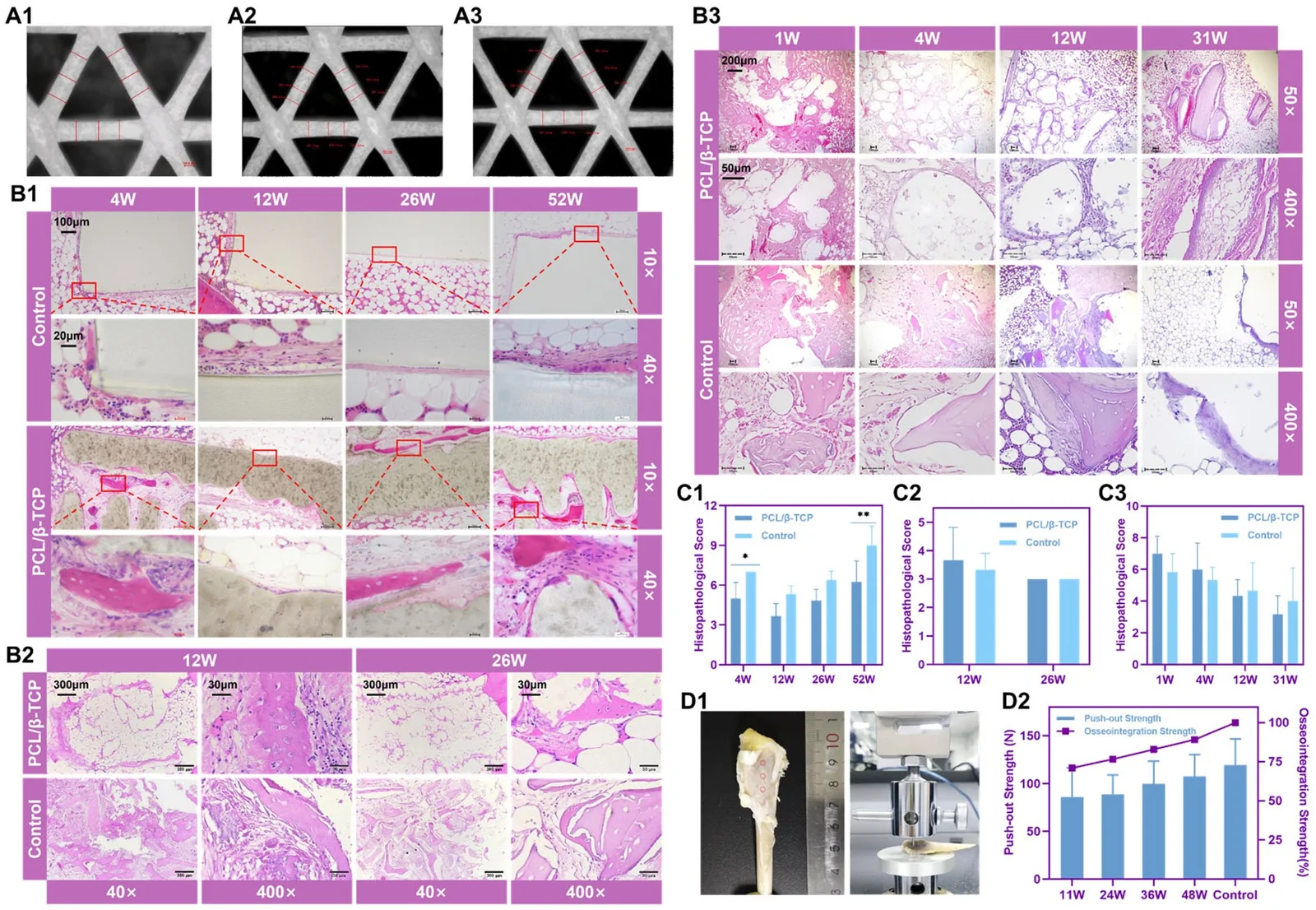
Degradation results of three stages *in vivo*. (**A1–A3**) Quantitative analysis of initial wire diameter of samples. (**B1–B3**) H&E staining section diagram. (C**1–C3**) Semi quantitative statistical diagram of histopathological score. (**D1**) Tibial tissue sample processing and bone push out test method, (**D2**) push-out strength and osseointegration strength of samples at different implantation cycles. (*P < 0.05, **P < 0.01, *n* = 4).

During the whole observation period from 1 to 31 weeks, the tissue response score caused by PCL/β-TCP composite scaffold was always maintained at the level of ‘minimal or no reaction’. The inflammatory reaction was mild at the initial stage of implantation. Although the number of giant cells and inflammatory cells increased slightly due to the activation of active clearance mechanism after 12 weeks, the overall reaction was still mild and controllable (Figure 5C3).

After the PCL/β-TCP composite scaffold was implanted into the rabbit tibia for 11 weeks, the bone bonding strength reached 71.07% of the normal tissue. With the increase of time, the bone bonding strength showed an overall upward trend, reaching 89.17% at 48 weeks, approaching the mechanical properties of normal bone tissue. It showed that the sample had good osseointegration with the surrounding bone tissue (Figure 5D).

### Experiment of canine oral cavity restoration

In the oral bone defect model, Micro-CT images showed that the PCL/β-TCP composite porous scaffold sample maintained the width and height of alveolar bone, while the width and height of alveolar bone at the implantation site in the control group were atrophic, and the proportion of black in the span of the bidirectional arrow was higher than that of the test sample (Figure 6B and C). The filling effect of PCL/β-TCP group and control group after 12 weeks of implantation was similar, and there was no significant difference in the quality of new bone. After 26 weeks of implantation, Micro-CT data showed that the BV/TV of PCL/β-TCP group was 46.93 ± 5.36%, which was significantly higher than that of the control group (37.08 ± 8.39%). The BMD of PCL/β-TCP group was 0.401 ± 0.012 mg/cm^3^, which was significantly higher than that of the control group (0.338 ± 0.023 mg/cm^3^). PCL/β-TCP group formed a large number of high-density mineralized bone, and the osteogenic quality, trabecular thickness and quantity were close to those of autologous bone (Figure 6D–G). The results of bone height and bone width measurement at 26 weeks showed that the control group had obvious collapse at the top of the extraction socket, and the boundary between bone powder and new bone was unclear, which could not accurately distinguish the new bone area. In the PCL/β-TCP group, no obvious atrophy was observed at the top of the extraction socket, and there was significant new bone ingrowth in the scaffold filled area. Compared with the control group, PCL/β-TCP group maintained the width (3.91 ± 0.15 mm) and height (3.78 ± 0.17 mm) of alveolar bone better, which was significantly higher than the width (2.68 ± 0.32 mm) and height (2.45 ± 2.24 mm) of the control group, and had no obvious implant stimulation response, with excellent biological safety (Figure 6H).

**Figure 6:**
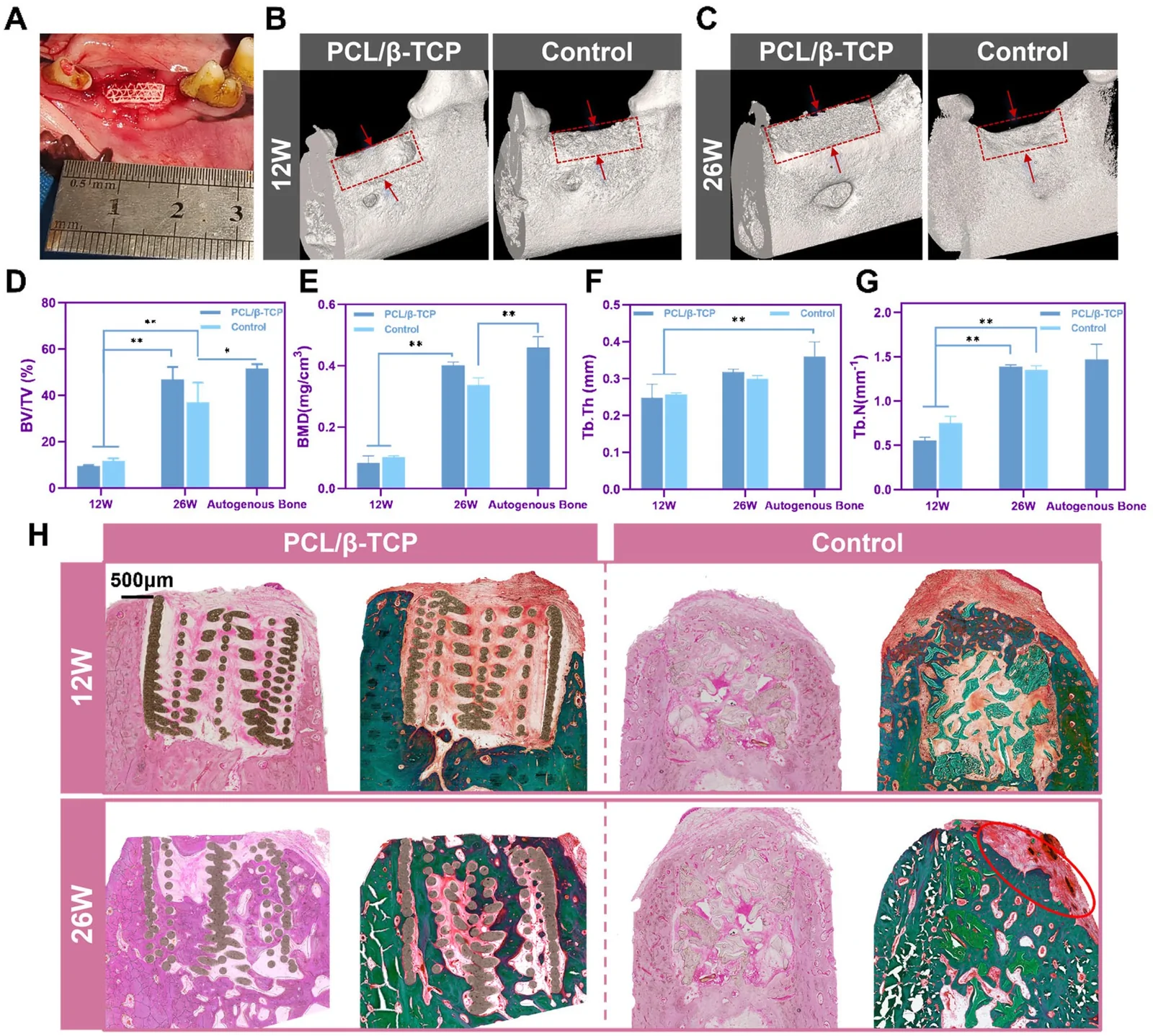
Experimental results of canine oral cavity repair. (**A**) Photo of molding position. (**B, C**) Micro CT images of oral bone defects at 12 and 26 weeks. The red dotted line is the defect location, and the two-way arrow indicates the location with the most alveolar bone atrophy. (**D–G**) Statistical chart of BV/TV, BMD, Tb.Th and Tb.N analysis of oral bone. (**H**) H&E and Goldner’s trichrome staining sections after oral bone defect implantation at 12 and 26 weeks; Goldner’s trichrome staining showed that the osteoid was orange, the mineralized bone was dark green and the circle marked the material collapse area. (*P < 0.05, **P < 0.01, *n* = 3).

### Degradation kinetics analysis

The degradation theory of PCL follows the first-order degradation dynamic equation [29, 30]. According to the research and test data, the number average molecular weight data of the degradation of composite scaffold products *in vivo* and *in vitro* were taken as the natural logarithm, as shown in Figure 7A–C. Firstly, the molecular weight decline has a highly linear relationship with time (*R*^2^ > 0.96). According to the linear regression equation, based on the first-order reaction kinetic model fitting, the three curves have the same law in the stage dominated by hydrolysis. The degradation rate constants of real-time, accelerated and *in vivo* degradation of the product are k_real-time_=7.78543 × 1 0 ^−9^, k_accelerate_=3.93567 × 1 0 ^−8^ and k_*in vivo*_=2.23699 × 1 0 ^−8^, respectively. The highly linear relationship between *in vivo* and *in vitro* degradation curves (R² > 0.96) confirmed the regularity of degradation process (Figure 7D) [31].

**Figure 7:**
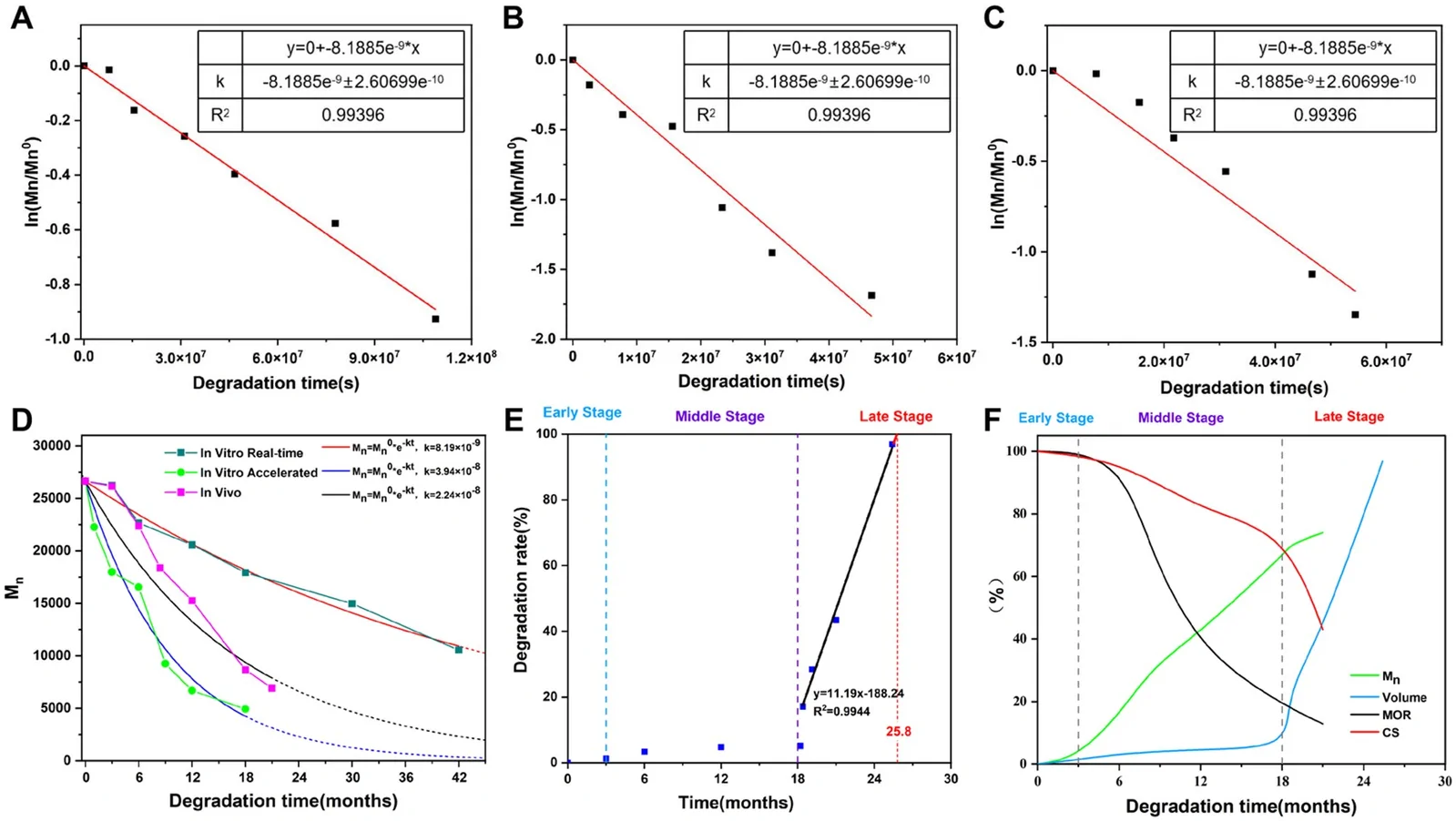
Degradation kinetics analysis. (**A–C**) Linear regression equations of *in vitro* real-time degradation (**A**), *in vitro* accelerated degradation (**B**) and *in vivo* degradation (**C**) versus time. (**D**) Fitting curve of degradation number average molecular weight and time *in vivo* and *in vitro*. (**E**) Change of degradation rate in the whole cycle. (**F**) Changes in molecular weight, volume, bending strength (MOR) and compressive strength (CS).

Based on the *in vivo* and *in vitro* molecular weight and degradation rate (Figure 7E) data and mechanical data (Figure 7F), the degradation of PCL/β-TCP composite scaffold products showed a clear three-stage rule, with a total cycle of about 26 months. The first stage of degradation (0–3 months): the core feature was the slow decline of molecular weight (about 1.8% molecular weight loss in 3 months *in vivo*), without significant volume loss. The mechanical properties and three-dimensional spatial structure were well maintained, and the compressive strength even increased slightly due to the increase of crystallinity. In the middle of the second stage of degradation (∼3–18 months): the molecular weight decreased faster (to 8664 Da in 18 months), and the volume loss started slowly (5.13% at 18 months). The mechanical properties began to decline, but it could still provide effective support for tissue regeneration and stability of three-dimensional osteogenic space within a certain period of time (the compressive properties did not decline in the first 18 months of real-time degradation, and the flexural properties decreased by 28.3% in 6 months). In the late stage of three-stage degradation (18 months—complete degradation): the molecular weight decreased to the low platform stage (<10 kDa), and the volume loss became the dominant mechanism, with cell-mediated phagocytic clearance as the main mechanism. The volume loss rate showed a highly linear growth (R² > 0.99), reaching 96.93% at 25 months and finally completed safe removal in about 26 months. It is important to note that the 26-month complete degradation cycle concluded in this study is specifically applicable to the tested 3D-printed scaffolds with an average strut diameter of ∼220 μm and high interconnected porosity. Because PCL degrades via a typical bulk erosion mechanism, the physiological fluid rapidly permeates the porous network. Consequently, the time required for the molecular weight to decline to the critical threshold (M_n_ < 10 kDa, marking the onset of the third stage) is governed by uniform chemical hydrolysis and remains fundamentally constant, irrespective of the scaffold’s macroscopic dimensions.

## Discussion

At present, the degradation cycle of bone implant or oral related products made of absorbable synthetic polymer materials is expected to be about 2–3 years, which is relatively long. In order to accelerate the observation of the degradation law of the declared product, the existing relevant industry standards mentioned that in order to observe the late stage events after implantation more quickly, the implantation of partially degraded materials *in vitro* could be considered. Therefore, we suggested that the degradation test of relevant products could be divided into three stages: early stage, middle stage and late stage [32–34]. Furthermore, while the ISO 10993-13 standard provides foundational guidelines for executing *in vitro* accelerated degradation testing, it lacks an operational framework for systematically correlating these *in vitro* findings with long-term *in vivo* biological responses. The uniqueness of our three-stage *in vivo* relay strategy lies in utilizing pre-aged samples to construct a seamless *in vivo* temporal continuum. By confirming the molecular weight and histopathological overlap at the stage transition nodes, this strategy goes beyond the standard by vastly reducing animal study times, thereby addressing current regulatory bottlenecks and adhering to the 3Rs principles of animal welfare.

In this study, *in vitro* studies accurately revealed the chemical mechanism (dominated by ester bond hydrolysis) and kinetic law (three-stage characteristics) of material degradation by controlling variables. First, *in vitro* chemical analysis confirmed that its degradation followed the classic bulk erosion hydrolysis mechanism of polycaprolactone, which is the chemical basis of all predictions [35]. Secondly, the data of molecular weight decline *in vivo* and *in vitro* are perfectly consistent with the first-order reaction kinetic model, which is not only an observation, but also a mathematically verified and quantitatively predictable law; Finally, through the stage overlap verification, that is, based on the change rule of molecular weight degradation obtained from the *in vitro* study, the *in vivo* study realized the complete coverage of the *in vivo* degradation process up to 109 weeks through the three-stage relay design (initial sample, accelerated sample for 6 months and accelerated sample for 9 months). Molecular weight status of implants at each stage, e.g. M_n_=15 266 Da at 52 weeks *in vivo* ≈ M_n_=16 551 Da at 6 months after accelerated degradation *in vitro*. After accelerated degradation for 6 months, the implantation *in vivo* for 26 weeks, e.g., M_n _= 8664 Da ≈ *in vitro* accelerated degradation for 9 months (M_n _= 9251 Da) can effectively overlap, which strongly proves that the *in vitro* model can reliably predict the behavior *in vivo*, and ensure the preciseness of research design and the continuity of data. In addition, the macro degradation rate (volume/mass loss) is highly consistent with the micro molecular weight decline, crystallinity change and mechanical property degradation, which is in line with the principles of materials science. Thus, we provide a closed-loop evidence chain for the predictable degradation behavior of materials.

A critical assumption of our relay strategy is that the *in vivo* degradation kinetics and host responses of *in vitro* pre-aged samples are identical to those of pristine samples naturally degraded to the same molecular weight. This premise is strongly supported by the two-stage degradation mechanism of PCL [31]. The initial degradation phase is a purely physicochemical bulk hydrolysis that remains identical whether in PBS or physiological fluid. Subsequent cell-mediated clearance is only triggered when the polymer fragments and M_n_ drops below a critical threshold (<10 kDa), recognizing the material’s current physical state rather than its hydrolytic history [36]. This theoretical assumption is robustly validated by our empirical data at the overlap node: pristine samples after 52 weeks *in vivo* (M_n_ = 15 266 Da) and 6-month *in vitro* accelerated samples (M_n_ = 16 551 Da) exhibited nearly identical histopathological scores and macrophage engagement, confirming that prior *in vitro* hydrolysis does not artificially alter subsequent cell-mediated clearance pathways.

The results of degradation kinetics also indicate that at the early stage of degradation, (M_n_ > 10 000–5000 Da) conforms to the first-order degradation kinetics, and the trends of real-time degradation and accelerated degradation are consistent, which strongly verifies the effectiveness of the accelerated model, and provides a core scientific basis for the strategy of using accelerated samples for *in vivo* experiments to predict long-term behavior. The acceleration factor AF (k_accelerate_/k_*in vivo*_)≈1.76 and the acceleration factor *in vitro* AF (k_accelerate_/k_real-time_ ≈ 5) show that the acceleration experiment can effectively simulate and predict the degradation behavior *in vivo*, which can be used for subsequent corresponding speculation. In the later stage of biodegradation, cells participate in the biodegradation, which starts when the polymer crystallinity is high and the molecular weight is low (M_n_ < 10 000–5000), and may be recognized by cells and eliminated by phagocytosis, and the clearance rate *in vivo* is accelerated.

PCL/β-TCP composite porous scaffold has the characteristics of rapid tissue chimerism. Although the hydrolysis point is exposed in the tissue, its degradation process is very slow, and obvious structural changes and mechanical properties will be reduced in the third stage *in vivo* [6, 37, 38]. Due to its inherent hydrophobic crystallinity, the pure PCL scaffold mainly depends on the slow hydrolysis process *in vivo*, and the degradation cycle is as long as several years, which limits its matching with the rhythm of bone repair [39, 40]. For PCL/β-TCP composite porous scaffolds, the degradation behavior changes significantly due to the introduction of β-TCP. At the early stage of implantation, β-TCP actively ‘dissolves’ and continuously releases calcium ions (Ca^2+^) and phosphate ions (PO_4_^3+^) to the local microenvironment. This process has two key roles: first, the alkaline phosphate ion effectively neutralizes the acidic degradation products produced by the breaking of the ester bond of PCL, significantly improves the autocatalytic hydrolysis effect usually aggravated by the decline of pH, thus delaying the breaking rate of the main chain of PCL from the chemical level; Second, the released ions promote the bone mineralization process of surrounding tissues, guide the new bone tissue to quickly fit into the cracks caused by micro degradation in the material, and endow the composite with excellent structural stability and mechanical support function for up to 18 months after implantation through the timely growth and replacement of tissues [12, 38, 41–43]. By comparing the degradation data of pure PCL scaffolds, we can also see that β-TCP increases the specific surface area of PCL matrix, accelerates the water penetration and ester bond hydrolysis, and improves the overall interpretation efficiency by enhancing the hydrophilicity and interface hydrolysis of PCL without changing the chemical degradation mechanism of PCL. More importantly, this β-TCP regulated and balanced degradation microenvironment avoids the trigger of acute and chronic inflammation and is the structural basis for the composite to show mild immune response and achieve good biocompatibility throughout the degradation cycle [44–47].

The release profile of β-TCP is a matrix-governed biphasic process inherently coupled with PCL degradation. Following the initial dissolution of surface-exposed particles, the sustained release of internal β-TCP is strictly rate-limited by the bulk erosion and structural loosening of the PCL matrix [48, 49]. The exposure kinetics of embedded β-TCP scale accurately with the acceleration factor. The β-TCP particles in the scaffold are completely encapsulated inside the PCL matrix, and there is no independent dissolution channel for inorganic phase ion release, which is completely dependent on the hydrolysis process of PCL ester bonds. The early degradation stage after implantation is mainly characterized by polymer hydrolysis reactions. Before the infiltration of macrophages and the phagocytic degradation of materials, PCL only undergoes simple ester bond hydrolysis. This chemical hydrolysis mechanism is completely consistent with the accelerated degradation system *in vitro*, and there are no additional consumption pathways mediated by enzymes or cells. Therefore, as mathematically validated by our IVIVC model showing highly correlated molecular weight and mass loss across both conditions (Figure 7), the synchronized β-TCP release behavior is reliably simulated by the accelerated model, which fundamentally drives the sustained osteogenesis and excellent long-term biocompatibility observed over 109 weeks *in vivo*.

In establishing the IVIVC model, our calculated acceleration factor (AF) for the PCL/β-TCP scaffold at 50°C was ∼5 relative to *in vitro* real-time degradation, and 1.76 relative to *in vivo* degradation. It is worth noting that this AF is significantly more moderate and stable compared to values typically reported for fast-degrading polymers like polylactic acid (PLA) and poly(lactic-co-glycolic acid) (PLGA). Studies have shown that when PLA or PLGA are subjected to elevated temperatures (e.g., 50–70°C), the testing conditions often cross their glass transition temperatures (*T_g_*, typically 45–65°C). This transition from a glassy to a rubbery state drastically increases water diffusivity and chain mobility, resulting in highly non-linear acceleration factors (often exceeding 10–50) and a potential shift in the fundamental degradation mechanisms [32, 50, 51]. In contrast, PCL has a very low *T_g_* (∼ −60°C), meaning it remains in the same physical rubbery state at both 37 and 50°C. Therefore, our 50°C accelerated protocol strictly follows Arrhenius kinetics without altering the bulk erosion mechanism, verifying that our IVIVC model for PCL-based scaffolds is highly predictable and scientifically sound.

From the overall pathological evaluation, this study covers the complete degradation process from the slow decline of molecular weight, maintenance of mechanical properties, to material fragmentation and cell phagocytosis. The histological response at each stage showed high consistency: no or very slight stimulation, accompanied by active bone integration and bone regeneration. The first stage: the phagocytosis of multinucleated giant cells was observed at 4 weeks after implantation, which confirmed the start of degradation, accompanied by the growth of fibrous vascular tissue and early new bone formation. The second stage: the number of multinucleated giant cells increased and the activity increased, which was a physiological clearance reaction, rather than a pathological inflammation. The new bone trabeculae distributed along the gap of the degraded material, and the process of bone integration was not disturbed. The third stage: the materials were rapidly fragmented and removed, and the local inflammatory cells only increased slightly and then subsided. At 109 weeks, most of the materials were replaced by new fibrous connective tissue and bone tissue to achieve smooth transition and functional regeneration. Throughout the whole degradation study, the most significant feature is that tissue growth and material degradation are carried out simultaneously. The new tissue (from fibrous and vascular tissue to bone tissue) effectively ‘crawls’ to replace the degraded material, and finally realizes the repair composed of autologous tissue without material residue. To evaluate the safety and efficacy of the PCL/β-TCP composite scaffold for alveolar bone defect repair, a multicenter, prospective, single-blind randomized controlled clinical study was conducted. The trial results confirmed the excellent performance of the product, successfully maintaining the height and width of the alveolar bone. In the Full Analysis Set (FAS), the effective bone fusion rate at 24 weeks was 83.67% in the treatment group and 81.63% in the control group, with no statistically significant difference (*P *> 0.05). However, in the Per Protocol Set (PPS), the bone fusion rate in the treatment group reached 100%, significantly higher than the control group’s 90.91%, with a statistically significant difference (*P *< 0.05). These results not only validate that the 3D-printed PCL/β-TCP composite scaffold is non-inferior or even superior to existing traditional materials in terms of bone fusion efficacy, but also highlight its ability to achieve excellent and stable bone regeneration under strict adherence to the treatment protocol. This provides a new and reliable option for the precise repair of alveolar bone defects.

At present, there are still multiple obstacles to the systematic evaluation of long-term biodegradable biomaterials. The first is the limitation of evaluation methods. *In vitro* degradation models are often oversimplified and cannot accurately simulate the complex physiological environment and its dynamic changes *in vivo*. The reliability and scientificity of predicting long-term *in vivo* behavior are questioned, and there is always a gap between animal evaluation models and human real reactions. In addition, the time and cost required to reach the degradation end point that meets the review requirements are huge, and the complexity of degradation in composites due to the different physical and chemical properties of different phase compositions also makes regulators lack scientific evaluation tools and standards when reviewing such innovative products. They often use traditional review standards and require strict degradation end point data and safety reports, which has become an important bottleneck restricting the clinical transformation of such innovative devices [1, 52–55]. Therefore, the innovation of absorbable medical devices also needs the upgrading of scientific evaluation methods of supervision. Since the definition of steady state is based on safety and effectiveness indicators as an important reference, we will take the product used in this study as an example to explore its biological safety and effectiveness in the whole cycle degradation process.

From the perspective of effectiveness, we evaluated the functional effectiveness indicators of the composite scaffold product from three aspects. The first is the mechanical characteristics and structural integrity. *In vitro* data directly confirmed that the scaffold product can maintain 90% of its compression performance and 60% of its bending performance within 9 months, and maintain the stability of three-dimensional spatial structure for up to 18 months. Within the key function window period of 6–9 months, the mechanical performance decline rate meets (or even is better than) the preset design goal of ≤ 30%, which provides the core functional evidence for its role of temporary mechanical support. Combined with the bone push out experiment in animal studies, the rabbit bone defect model was pulled out for testing. After 6 months of critical period of bone healing, the scaffold with good surrounding tissue integration needs 29.39 ± 13.85 MPa force to be pushed out, and the bone fusion ratio is 76.64%, which proves the mechanical locking effect brought by tissue growth. Secondly, in terms of the effect of bone regeneration ability, combined with animal studies, histological evidence such as the formation of bridge callus and the distribution of new bone trabeculae along the scaffolds in the Beagle Dog oral and maxillofacial bone defect model, as well as the bone mass recovery and bone mineral density increase confirmed by imaging in clinical trials, jointly proved that the product successfully guided bone regeneration and created a qualified bone bed for implant repair. Finally, there is the matching of degradation and regeneration: the body erosion mode of ‘molecular weight reduction first, then mass loss’ is essentially a design that perfectly matches the ‘crawling replacement’ bone regeneration strategy. It ensures that the material degrades gradually with the growth of the tissue. To sum up, we believe that the composite scaffold product has achieved all the preset functional goals as an absorbable bone regeneration scaffold through the evaluation of the above three key elements.

Based on the perspective of biosafety, we conducted a prospective risk assessment. The first is safety. The concentration of the degradation product (caprolactone monomer) is always lower than the strict safety threshold of 10 ppm, and does not show a continuous accumulation trend. The observation data show that the product will further hydrolyze and open the ring, and finally enter the natural metabolic pathway, which chemically eliminates the risk of cumulative toxicity. From the perspective of biological safety, during the whole process of total degradation and absorption *in vivo*, the tissue response score was always maintained at the level of ‘no/very slight stimulation’, which showed that the host response was mild and controllable in both the early stage of degradation and the debris clearance period. In addition, we also conducted a long-term residual risk assessment, that is, the theoretical risk of ‘long-term incomplete elimination’. Animal data for more than two years showed that even if the material remained, it did not cause chronic inflammation, fibrous encapsulation or bone remodeling inhibition.

Throughout the degradation process, there is a continuous interaction between the implanted material and the body, especially the continuous changes in the interface between the material and the tissue, which is the key to the safety and effectiveness of the product, and also a risk factor that needs to be focused from the perspective of regulatory science. From the perspective of the degradation process of materials in this study, the degradation of materials and tissue ingrowth process are coordinated and synchronized, and the reaction is slightly controllable. New bone formation is continuously observed. The histopathological score changes in the first and second stages *in vivo* are relatively gentle, showing a trend of rising briefly at the initial stage and then gradually tending to be gentle. The highest point of the overall stimulation degree is in the degradation debris removal phase in the third stage, that is, the time point of 12 weeks after 9 months of accelerated degradation (the corresponding real-time implantation cycle *in vivo* is 90 weeks), but the overall immune response is still slightly controllable. For such products, the clinical effectiveness required by the mechanical audit department is generally about 6 months after implantation. However, from the case of this study, it can be seen that the critical period of clinical effectiveness does not coincide with the biological risk peak. The composite stent maintains good support performance within 18 months after implantation, but its immune response peak is after it, and the debris clearance period is about 22 months after implantation. After this critical time point, we believe that the degradation of the material has reached a stable state of tissue reaction, which can approximately replace the degradation end point. This also suggests that it is not necessarily sufficient for us to reach the end point of product effectiveness. For the judgment method of ‘tissue material steady state’ in the long-term degradation process of similar absorbable medical devices, we believe that the degradation cycle should be at least greater than the end point of effectiveness and the peak value of safety risk at the same time.

It is crucial to emphasize that the concept of a ‘risk peak’ during degradation is not specific to PCL/β-TCP scaffolds but represents a universal paradigm across the entire field of absorbable medical devices. Due to the dynamic nature of degradation, implantable materials inevitably transition from a mechanically intact state to structural disintegration, triggering distinct biological responses. For instance, classic fast-degrading polymers such as PLA and PLGA characteristically exhibit a well-documented ‘late risk peak’. Following a period of clinically silent bulk hydrolysis, these materials abruptly fragment, releasing a massive burst of acidic oligomers and highly crystalline debris. This rapid mass loss frequently triggers a delayed foreign body response or sterile inflammation months to years post-implantation [56]. Conversely, absorbable metals like magnesium alloys typically manifest an ‘early risk peak’. Immediately following implantation, rapid initial corrosion occurs, generating local hydrogen gas pockets and an alkaline microenvironment before a protective passivation layer can form, representing the highest risk to local tissue viability [57]. Furthermore, the classic bulk erosion theory dictates that all such polymers will undergo a sudden, non-linear mass loss phase [35]. Therefore, identifying the material-specific ‘risk peak’—whether it occurs early due to acute surface reactivity (e.g., metals) or late due to bulk fragmentation and macrophage-mediated clearance (e.g., polyesters)—is a universally applicable and essential scientific premise for defining ‘tissue-response homeostasis’ and ensuring the long-term safety of any biodegradable device.

Based on this, we have come to the definition of steady state. First, from the perspective of biological safety, the degradation products should be below the safety threshold without continuous accumulation risk. The tissue response of materials should have passed the biological safety peak (the typical peak includes the highest point of gradual rise of histological stimulation at the initial stage of implantation and the risk period of debris clearance at the end), and enter the stable period of histological response; Secondly, from the perspective of functional effectiveness, in addition to meeting the mechanical support within the critical functional window period (6–9 months), especially for the absorbable alveolar bone repair scaffold in this study, alveolar bone increment is of great significance for clinical repair effect. Therefore, the material should also meet the promotion of bone regeneration and ‘degradation regeneration’ adaptation in a long time, that is, provide sufficient and stable osteogenic environment for bone tissue for about 3–18 months. We defined the safety threshold as a histopathological score of ≤2.0 (minimal to no reaction) sustained after the peak degradation clearance phase, alongside local degradation product concentrations remaining below the cytotoxic threshold. For efficacy, we defined the threshold as the maintenance of ≥50% mechanical compressive strength during the critical 6–9-month bone healing window, coupled with significant continuous osteogenesis, and the monomer concentration of local degradation products is always lower than the safety threshold (e.g., <10 ppm). That is to say, if the steady state is taken as the key observation time point of material degradation, then this time point should at least meet the requirements that the biological safety risk is lower than the risk peak and the functional effectiveness reaches the expected design level of the material. This definition can be extended to different similar absorbable medical devices according to the actual clinical use environment, which can be used to assist in judging the critical observation period of product degradation and shorten the evaluation cycle, which is of great significance for the development of evaluation system of innovative products and the process of clinical transformation.

## Conclusion

Based on the evaluation system of absorbable medical devices, this study confirmed the influence mechanism of β-TCP on the degradation performance of PCL, and verified the safety and effectiveness of PCL/β-TCP composite porous alveolar bone repair scaffold material by using the *in vivo* and *in vitro* correlation degradation evaluation model. Based on this, the concept of tissue response steady state and the judgment method of safety and effectiveness risk peak were proposed, which provided key experimental evidence for the definition of steady state, and could be extended to systematically evaluate other similar tissue engineering products, so as to accelerate the clinical application of truly bioactive regenerative medicine products, and ensure the scientificity and preciseness of its regulatory review.
